# Proposed Risk of Bias Assessment Tool for *In Vitro* Antimicrobial Susceptibility Studies

**DOI:** 10.3390/pathogens15040396

**Published:** 2026-04-07

**Authors:** Matthew E. Falagas, Dimitrios Ragias, Dimitrios S. Kontogiannis, Laura T. Romanos, Paraskevi A. Farazi

**Affiliations:** 1 Alfa Institute of Biomedical Sciences (AIBS), Marousi, 151 23 Athens, Greece; d.ragias@aibs.gr (D.R.); l.romanos@aibs.gr (L.T.R.); 2 School of Medicine, European University Cyprus, 2404 Nicosia, Cyprus; e.farazi@euc.ac.cy; 3 Department of Medicine, Tufts University School of Medicine, Boston, MA 02111, USA

**Keywords:** risk of bias, contamination/cross-contamination bias, heterogeneity, measurement/observer bias, methodological bias, publication bias, reporting bias, selection bias

## Abstract

The assessment of risk of bias in systematic reviews and meta-analyses is crucial, as it indicates the accuracy of the synthesized and evaluated data and the validity of the presented results and conclusions. Until now, standardized tools for this purpose have been available only for clinical and animal studies, while adapted forms of these tools and novel ones have been proposed for *in vitro* and laboratory studies. However, none of them have been universally standardized so far. The apparent lack of a risk of bias assessment tool for systematic reviews of *in vitro* antimicrobial susceptibility testing studies constitutes a methodological flaw in these studies. To this end, we developed a risk of bias assessment tool for *in vitro* antimicrobial susceptibility testing studies. Our tool assesses the risk of bias across six domains: methodological bias, selection bias, preparation bias (including contamination/cross-contamination bias), measurement/observer bias, reporting and publication bias, and bias related to unreported funding and conflicts of interest. The tool evaluates a total of 16 specific criteria. The risk of bias is graded as low, moderate, or high for each evaluated criterion. The proposed risk of bias assessment tool was tested in a pilot validation study of ten relevant studies by two reviewers independently. We believe that the use of the proposed risk of bias assessment tool will increase the methodological strength of systematic reviews and meta-analyses of *in vitro* antimicrobial susceptibility testing studies.

## 1. Introduction

A critical component of conducting a systematic review or meta-analysis is assessing the risk of bias in each of the included relevant original studies. This refers to the likelihood that the results presented are biased, which could compromise the transparency, consistency, and trustworthiness of the review/meta-analysis [1,2,3]. Based on the findings of this assessment, the authors decide whether to include or not a specific study in their systematic review or meta-analysis. When included, authors make statements regarding the methodological limitations and the interpretation of the results. Additionally, readers of systematic reviews or meta-analyses can make more informed decisions, as they have access to detailed methodological information about each original study [4]. In systematic reviews and meta-analyses of *in vitro* antimicrobial susceptibility studies, however, risk of bias assessment is not usually applied. This could be attributed to the lack of standardized tools and to the use of adapted versions of the available tools developed for other types of studies, including clinical and *in vitro* studies [5]. Our study aims to fill this gap by proposing a newly developed, validated risk of bias tool explicitly developed for *in vitro* antimicrobial susceptibility studies.

## 2. Main Types of Bias

There are several types of biases that authors should consider when conducting systematic reviews or meta-analyses. Both clinical [randomized controlled trials (RCTs), non-randomized interventional trials, and observational studies of various methodological types] and preclinical studies, including animal and *in vitro* studies, may be biased by the selection of reported results (reporting bias). Additionally, preferential reporting of positive results may occur in all types of studies (publication bias). However, other biases may vary depending on the type of study. More specifically, RCTs may be subject to bias due to randomization procedures, deviations from the intended interventions, missing outcome data, and issues with outcome measurement [6,7]. Non-randomized studies of interventions and observational studies may also be subject to bias related to confounding, classification of interventions, participant selection, and missing data [8,9]. The *in vitro* studies, including antimicrobial susceptibility testing studies, may also be subject to various types of bias, such as preparation bias, methodological bias, measurement (or observer) bias, publication bias, and contamination (or cross-contamination) bias [10,11,12,13].

## 3. Risk of Bias Assessment Tools for Interventional Studies

Several tools are available for assessing the risk of bias in clinical studies. The “Revised Cochrane risk of bias tool for randomized trials” (RoB 2), designed to evaluate risk of bias in randomized trials, includes templates for randomized parallel-group trials, cluster-randomized parallel-group trials, and randomized crossover trials. This tool has five evaluation domains, which are applied in each study. Specifically, these domains address “bias arising from the randomization of each study”, “deviations from the intended interventions”, “missing outcome data”, “outcome measurement”, and the “selection of reported results” [7].

The “Risk Of Bias In Non-randomized Studies-of Interventions version 2” (ROBINS-I V2) tool is designed to evaluate the risk of bias in non-randomized, cohort studies. The evaluation is based on seven different domains, specifically, “bias due to confounding,” “bias in selection of participants into the study,” “bias in classification of interventions,” “bias due to deviations from intended interventions,” “bias due to missing data,” “bias in measurement of outcomes,” and “bias in selection of the reported result.” For each of these domains, ROBINS-I employs a series of “signaling questions” to gather information about the study, and the authors then determine the risk of bias as “low,” “moderate,” “serious,” or “critical.” These domain-level judgements contribute to the overall risk of bias evaluation [8].

## 4. Risk of Bias Assessment Tools for Non-Interventional, Observational Studies

A modeled version of the ROBINS-I tool, namely the “Risk Of Bias In Non-randomized Studies–of Exposures” (ROBINS-E) tool, was developed for assessing the risk of bias in non-interventional, observational studies of exposure [14]. In this context, this tool differs from ROBINS-I in that it includes domains evaluating the risk of bias in the measurement of exposure and departures from exposure, rather than biases related to the intervention.

## 5. Risk of Bias Assessment Tools for Pre-Clinical (*In Vitro* and Animal) Studies

### 5.1. Adapted SYRCLE

The Adapted Systematic Review Center for Laboratory Animal Experimentation (Adapted SYRCLE) risk of bias tool is an adaptation of the Cochrane risk of bias tool, which is typically used for randomized controlled trials in human clinical research. In SYRCLE, this Cochrane framework is being readjusted for use in animal studies; when adapted, it can also be applied to *in vitro* studies [15,16].

### 5.2. In Vitro Experimental Studies Internal Validity (INVITES-IN) Tool

The *in vitro* experimental studies internal validity (INVITES-IN) tool is a risk of bias assessment instrument specifically designed to evaluate the internal validity of *in vitro* studies, particularly those involving cell cultures. It is structured around signaling questions that are related to the design and conduct of the studies. A beta version of this tool has been released, covering areas such as “sample selection and allocation,” “blinding of personnel and outcome assessors,” “handling of missing data,” and “selective reporting of results.” The final, publicly released domains will be detailed upon the tool’s complete publication [17].

### 5.3. Risk of Bias Tool for Clinical Laboratory Studies (Latitudes Network)

The risk of bias tool for clinical laboratory studies published by the Latitudes Network is a pilot tool developed by consensus to validate clinical laboratory studies, including both comparative and single-arm designs. It is available via the Latitudes Network. It assesses these studies for bias using three main domains: “collection and handling of samples,” “experimental methods,” and “reporting of results.” Further development and validation are needed for the broader application of this tool [18].

### 5.4. The Risk of Bias Tool for Pre-Clinical Dental Materials Research (RoBDEMAT)

The RoBDEMAT tool is a specialized tool and guideline designed to support the reporting of pre-clinical dental materials research investigations and to improve the assessment of systematic reviews in the field of dental materials. It contains four domains: “bias related to planning and allocation,” “specimen preparation,” “outcome assessment,” and “data treatment and outcome reporting.” It includes nine items pertaining to different sources of bias within those domains, signaling questions, and a guide that could be used for risk of bias judgment. The sources of bias are, namely, “control group,” “randomization of samples,” “standardization of samples and materials,” “identical experimental conditions across groups,” “adequate and standardized testing procedures and outcomes,” “blinding of the test operator,” “statistical analysis,” and “reporting study outcomes.” This tool is intended for use by two reviewers working independently. An individual RoBDEMAT should be completed for each study included in a systematic review. A third reviewer should be available for conflict resolution [19].

## 6. Quality Assessment Tools for Research

Quality assessment tools for various types of studies, including *in vitro* antimicrobial susceptibility testing studies, are based on a detailed checklist that evaluates the precision in the design, conduct, and reporting of these types of studies. Their use has been suggested to assess the possible risk of bias in *in vitro* antimicrobial susceptibility testing studies. However, they go beyond the risk of bias assessment level, including a large number of items. Such tools are the Joanna Briggs Institute (JBI) Critical Appraisal Tools, the Newcastle-Ottawa scale, and the CASP (Critical Appraisal Skills Programme) tools [20,21,22,23]. Additionally, the QUIN (Quality *in vitro*) tool was developed to evaluate the methodological quality and risk of bias in *in vitro* studies in dentistry [24].

## 7. Need for Development of a Risk of Bias Assessment Tool for *In Vitro* Antimicrobial Susceptibility Studies

The assessment of risk of bias is less frequent in systematic reviews of *in vitro* studies, including *in vitro* antimicrobial susceptibility testing studies, than in clinical studies. This has become evident to our research team recently during the conduct of relevant systematic reviews of *in vitro* antimicrobial susceptibility testing studies, specifically the evaluation of resistance to ceftazidime-enmetazobactam, aztreonam-avibactam, and sulbactam-durlobactam [25]. However, several sources of heterogeneity that are potentially related to bias require the attention of authors of systematic reviews and meta-analyses of such studies. Thus, there is a need to develop a risk of bias assessment tool to be used explicitly in *in vitro* antimicrobial susceptibility studies.

Furthermore, the existing risk of bias tools for *in vitro* studies fail to evaluate the risk of bias in *in vitro* antimicrobial susceptibility testing studies. In this context, we performed and presented a comparison between commonly used risk of bias tools for *in vitro* studies, such as INVITES-IN, QUIN, and RoBDEMAT, and their inadequacy to evaluate the risk of bias in *in vitro* antimicrobial susceptibility testing studies (Table 1).

## 8. Methodological Issues of *In Vitro* Antimicrobial Susceptibility Testing Studies

### 8.1. Breakpoints

A primary source of variation frequently stems from the lack of standardized resistance breakpoints. For instance, when resistance breakpoints for an antibiotic combination have not been clearly defined by any relevant organization, such as the European Committee on Antimicrobial Susceptibility Testing (EUCAST), Food and Drug Administration (FDA), or Clinical and Laboratory Standards Institute (CLSI), authors typically use breakpoints of one of the constituents of the combination or prospective ones, usually based on available pharmacokinetic/pharmacodynamic (PK/PD) data. In these cases, the minimum inhibitory concentration (MIC) used to characterize a pathogen as susceptible or resistant varies between articles, hindering our ability to reach a definitive conclusion about the isolates’ resistance to the drug in question. Even when breakpoints have been defined, discrepancies often persist between MIC values reported by the relevant organizations. This way, it is difficult to make a fair comparison of resistance between two studies that used breakpoints defined by different organizations.

In some articles, tables presenting MIC_50_ and MIC_90_ values may include additional data on the distribution of the *in vitro* antimicrobial susceptibility testing results of the studied isolates. However, even in this case, it is sometimes impossible to draw conclusions about the percentage of resistant isolates, as some breakpoint criteria include a third category, defined as intermediate resistance/susceptibility (abbreviated as I by CLSI), susceptible with increased exposure (abbreviated also as I by EUCAST), or susceptible dose-dependent (SDD) [26,27].

### 8.2. Proposed Risk of Bias Assessment Tool in In Vitro Antimicrobial Susceptibility Studies

A universally accepted risk of bias assessment tool specifically designed for *in vitro* antimicrobial susceptibility testing studies has not yet been available. In Table 2, we present the proposed risk of bias assessment tool in *in vitro* antimicrobial susceptibility studies. It encompasses criteria in six domains, each associated with a specific type of bias, as discussed below. Each domain consists of several specific criteria, which are items that assess the risk of bias for the relevant type of bias within the domain. A total of 16 specific criteria have been proposed to adequately assess the risk of bias in *in vitro* antimicrobial susceptibility testing across 6 study domains, as discussed below.

**Scoring of each criterion**

High: High risk of bias (or not reported relevant data for assessment)
Moderate: Moderate risk of bias
Low: Low risk of bias

**Scoring of each domain**

High: when at least one criterion in the domain is scored as “high” risk of assessment, or >50% of the criteria in each domain are scored as “moderate” risk of bias
Moderate: when ≤50% of the criteria in each domain are scored as “moderate” risk of bias (and none as “high” risk of bias)
Low: when all of the criteria in each domain are scored as “low” risk of bias

**Total interpretation of the scoring**

High risk of bias: when at least one of the domains is scored as “high” risk of bias or >50% of the domains (i.e., at least 4 from 6 domains) are scored as “moderate” risk of bias
Moderate risk of bias: when at least one of the domains is scored as “moderate” risk of bias (and none as “high” risk of bias) or ≤50% of the domains (i.e., up to 3 from 6 domains) are scored as “moderate” risk of bias
Low risk of bias: when all of the domains are scored as “low” risk of bias

### 8.3. Methodological Bias

A detailed design and presentation of a protocol, adhering to a specific methodology, are essential components that support accuracy in *in vitro* antimicrobial susceptibility testing studies. Deviations from a predefined plan would pose a serious risk of bias. Internationally recognized and standardized guidelines and protocols (e.g., CLSI and EUCAST guidelines) have provided thorough guidance on the standard operating procedures for the various steps to be performed and reported in the methodological design of the study [28,29]. These include justified strain selection methods and media, documentation of calibrated and properly used media and equipment, detailed stock preparation, sterilization, storage, and standardization, inoculum standardization, incubation conditions, disc diffusion and MIC methods, measurement of inhibition zone diameters and interpretation, along with interpretation criteria, as well as quality control with the use of defined control strains. Methodologically detailed studies, based on these guidelines, are less likely to be biased. Finally, the use of appropriate statistical models and analyses further minimizes the risk of methodological bias. The justification of the methods used, if applicable, can support the reason for their selection.

### 8.4. Selection Bias

The application of this detailed methodology begins with the proper selection of isolates. Subsequently, the assessment for possible selection bias in *in vitro* antimicrobial susceptibility testing studies should be conducted. In this context, studies are primarily evaluated based on the detailed presentation of their inclusion and exclusion criteria. Additionally, studies are assessed regarding the isolate selection process, which can be either random (selecting isolates from a larger pool) or consecutive (selecting isolates in the order they are collected). The assessment should be based on the detailed report of the source and characteristics of the tested isolates, including body fluid (e.g., blood, urine, sputum) or tissue source, antimicrobial resistance phenotype and genotype (if available), collection date, and clinical relevance.

Moreover, the sample size and diversity of isolates should be evaluated for their adequacy and representativeness of real-world settings. Typically, 30 isolates are commonly considered the minimum threshold for sufficient basic reporting. In comparison, 100 or more isolates are the recommended standard for species-level testing, providing adequate statistical confidence in determining resistance rates [30]. The term “diversity of isolates” refers to both clinical and geographic diversity, encompassing multiple sample types, from different hospitals, regions, or patient populations, a range of resistance phenotypes, and both susceptible and resistant isolates. Selection bias may be present in studies using only reference strains [e.g., American Type Culture Collection (ATCC)] or stored or archived selected isolates. The use of selected strains (with specific characteristics, such as carbapenem-resistant isolates) should be explicitly justified when applicable. The inclusion of both clinical and reference strains (for quality control or validation) could minimize selection bias.

### 8.5. Preparation Bias (Including Contamination/Cross-Contamination Bias)

The reported use of proper materials and precise (calibrated and validated) equipment and methods, in accordance with EUCAST and CLSI guidelines and standard operating procedures for every step, is indicative of a low risk of biased results. In this context, the risk of bias regarding the preparation process for conducting an *in vitro* antimicrobial susceptibility testing study should also be assessed. Furthermore, contamination or cross-contamination before or during preparation may result in falsified results. Regarding this, an *in vitro* antimicrobial susceptibility testing study should be evaluated for risk of bias related to contamination.

First, the risk of preparation bias in the inoculum preparation process, its standardization, and inoculation procedure should be assessed. Regarding the preparation of the inoculum, the reported use of single, fresh colonies from a streak plate or a glycerol stock verified as pure is suggestive of a low risk of bias. This can also be enhanced by a report on limited subculturing onto non-selective media for purification, aiming to minimize both phenotypic and genotypic changes. On the other hand, the reported use of overgrown, atypical, mixed, or cultures from old plates poses a high risk.

Furthermore, when preparing the inocula, the reported sequential handling of isolates, one at a time, is necessary to minimize risk for contamination. Furthermore, the reported practice of checking subcultures for morphological consistency before susceptibility testing suggests a low risk.

After that, the reported use of high-purity antimicrobial standards by known, verified suppliers would minimize the risk.

Preparation bias may also be present when solvents for antibiotic dissolution are incompatible or improperly stored, leading to antibiotic degradation, as well as when the standardization of dilution procedures and media is uncertain. A low bias is suggested when soluble, stable, and compatible solvents and diluents are used, according to the CLSI or EUCAST standard laboratory guidelines. Moreover, a further report on the potency verification of antimicrobial batches by running positive and negative controls for each antibiotic tested, using appropriate quality control reference strains, would help minimize this risk. Apart from antimicrobials, the use of negative controls and uninoculated media can also validate the equipment and media, supporting a low risk of contamination. In cases of unexpected or borderline results, the reported repeat of preparation according to guidelines is suggestive of accuracy in the process and a low risk of preparation and contamination bias.

In addition, a high risk of preparation bias could also be posed by the lack of proper stock solutions (e.g., Mueller-Hinton agar and broth for most bacteria), which are not accurately prepared, sterilized, and stocked according to standard protocols, as well as the use of precise equipment for these purposes. In this context, pH and composition of media should reportedly fall within acceptable ranges, including correct cation concentrations, and contamination-protected conditions. Additionally, adequately and clearly labeled materials, including preparation dates, concentrations, storage instructions, and expiration dates, can help ensure proper use. Along with these, information on the incubation, including the use of calibrated equipment (pipettes, incubators, and timers) and proper conditions, should be evaluated.

The incubation process could be assessed for contamination bias by visual check by the investigators for mixed colony types or unexpected turbidity. In cases of reported possible contamination, such as inconsistencies in results, descriptive documentation of the media and methods used to identify the contamination source would suggest a low risk of contamination bias in the results. In these cases, the repeat of the entire procedure from a fresh culture is necessary to be clearly stated.

Finally, the reported practice of aseptic methods and techniques, utilizing properly labeled, stored, and sterilized materials (isolates, drugs, solvents, stocks, media, tubes, and plates) and equipment, as well as sterilized conditions throughout the entire process, is essential in these studies

### 8.6. Measurement/Observer Bias

Proper preparation is the first essential for ensuring measurement accuracy. The risk of biased results due to the observers conducting the measurements is the next point that should be assessed. To minimize the risk of bias in measurements, the results should be read by observers trained in standard *in vitro* antimicrobial susceptibility testing procedures, blinded to isolate identity, antibiotics, or expected results, including reference isolates or isolates for which susceptibility is known before testing. A comparison and verification of results by two or more independent observers could increase their validity.

For the performance of zone and MIC measurements, a variety of standardized reading techniques using defined guidelines for interpretation—the standard interpretation criteria—have been published by CLSI and EUCAST. Detailed information on the measurement process, performed using calibrated tools, could further support the accuracy of the results.

Furthermore, presenting the complete documentation of results, all results of repeat experiments, with averages and standard deviations, promotes reproducibility and transparency and poses a lower risk of bias. Again, in cases of unexpected or borderline results and/or discrepancies between measurements exceeding a threshold (e.g., ±2 mm zone or ±1 dilution MIC), the measurements should be repeated and reviewed. Finally, verifying the measurement process with reference strains for calibration falling within published quality control ranges could further minimize the risk.

### 8.7. Reporting and Publication Bias

To minimize the risk of reporting bias, a detailed report is essential that includes all definitions of the terms used, the tested isolates, media, methods, and results, including both positive and negative results, as well as those that are either expected based on previously published scientific data or unexpected, such as unforeseen MICs or deviations. In this direction, the reported use of established reporting guidelines, especially those proposed by EUCAST and CLSI, along with a structured checklist, suggests a low risk of bias. Moreover, the selections made in the study, such as strain and testing methods selection and exclusion, should be reasoned in detail.

On the other hand, under-reporting, selective reporting of only favorable and statistically significant results, or reporting high efficacy based on limited data and/or without statistical evidence, creates a serious bias. The presentation of raw data, along with the results of repeat experiments, including MIC or zone values, as well as averages and standard deviations, for each strain where appropriate, in Supplementary Files and/or open repositories, even if not obligatory, further supports a low risk of under-reporting.

Additionally, public registration and availability of protocols are indicative of a low risk for publication bias. Pre-registration, although uncommon in *in vitro* studies, can be considered, as some journals and platforms allow it (e.g., OSF, protocols.io). Moreover, a clear statement of commitment to publish the results, regardless of whether they are considered positive or negative, included in the protocol could help decrease the risk of publication bias.

### 8.8. Funding and Conflicts of Interest

Finally, possible economic or other interests, such as funding sources, as well as potential conflicts of interest that could bias the results, should be clearly disclosed. Unreported funding sources and unclear conflicts of interest suggest the potential for biased results.

## 9. Scoring and Interpretation

In Table 2, we also present the scoring for each criterion and domain, along with the overall interpretation of the proposed risk of bias assessment tool for *in vitro* antimicrobial susceptibility studies. Each specific criterion can be scored as “low”, “moderate”, or “high” risk of bias based on whether the study includes the characteristics mentioned above. We suggest that, when no relevant data on a specific criterion are reported for assessment, this should be scored as “high” risk of bias.

### Usefulness of the Proposed Risk of Bias Assessment Tool

Until now, systematic reviews of *in vitro* antimicrobial susceptibility testing studies have lacked a standardized tool for assessing the risk of bias in the included studies. A small number of such tools have been published, including ones used for clinical and animal studies, which have been adapted for *in vitro* studies. These tools aim to fill this gap; however, these studies extend to a comprehensive spectrum of expertise, and such tools cannot be universally applied to all types of *in vitro* studies. Moreover, the use of quality assessment tools, despite their detailed structures that extend beyond typical risk of bias tools, is extensive and not easy to apply in systematic reviews. In this context, we proposed a detailed risk of bias assessment tool for *in vitro* antimicrobial susceptibility testing studies, comprising six domains and 16 specific criteria. We consider that this tool can be easily applied when conducting systematic reviews or meta-analyses, as the specific criteria have been put with brevity and clarity. In this manuscript, the descriptive elaboration on these criteria aims to further help investigators in making their decisions.

In this context, we present one fully worked example of the domain-level and overall scoring of the proposed risk of bias assessment tool for *in vitro* antimicrobial susceptibility testing studies (Supplementary File S1).

## 10. Pilot Validation Study

We retrieved the 10 most recent articles on *in vitro* antimicrobial susceptibility studies archived in PubMed [31,32,33,34,35,36,37,38,39,40]. The search was performed on 15 October 2025. Specifically, we used pre-defined exclusion criteria. These included systematic reviews, case reports, case series with 10 patients or fewer, and studies involving pathogens isolated solely from animals. In articles reporting isolated pathogens from both animals and humans, we assessed the aspects of *in vitro* antimicrobial susceptibility testing performed only for those isolated from humans. Furthermore, the included articles were eligible for assessment if they reported details on the performance of *in vitro* antimicrobial susceptibility testing. Throughout the article selection process, we came across studies that performed *in vitro* antimicrobial susceptibility testing only by reference without further elaboration on the various steps and procedures. For this reason, those articles could not be evaluated according to the specific criteria of our tool.

Two reviewers independently used our proposed risk of bias assessment tool for *in vitro* antimicrobial susceptibility studies to evaluate and grade the studies according to the specific domains and criteria in the tool. Their reports are presented in Supplementary Tables S1 and S2, and the comparison of the results between the two reviewers is presented in Supplementary Table S3. During the validation process, we identified a critical issue. We observed that, while most studies followed CLSI or EUCAST guidelines for interpreting resistance status, a considerable proportion did not perform replicate experiments or exhibited other methodological deviations from these guidelines.

We acknowledge that repeating all procedures, especially *in vitro* antimicrobial susceptibility testing of a large number of pathogens, may be difficult due to economic constraints and excessive workload. However, this is a critical point in the CLSI standard operating procedures, and the lack of experimental replication could affect the accuracy of the results. In our evaluation of articles, this criterion alone led to the majority being characterized as high risk of bias. Given the importance of replicating experiments, as recommended by relevant organizations, we suggest that studies that do not meet this criterion be assessed as having a high risk of bias. This is especially important in cases of unexpected and/or borderline results.

Apart from that, based on the results from our pilot validation, we showed that in *in vitro* antimicrobial susceptibility testing studies, critical points may not be properly performed or adequately described. This resulted in the evaluation of a high percentage of bias in these studies and consequently in the literature. The fact that all studies were scored as having “high risk of bias” could be attributed to the lack, primarily, of replicate experiments, and secondly, of such points in the performance of various standard operating procedures or their presentation that could give rise to risk of bias issues.

According to the pilot validation, the agreement among reviewers’ domain assessments ranged from 66.7% (agreement in 4 out of 6 domains) to 83.3% (agreement in 5 out of 6 domains). Furthermore, we assessed the inter-rater reliability of the proposed antimicrobial susceptibility testing tool. The overall inter-reviewer agreement across all assessed domains and studies was substantial. The unweighted Cohen’s kappa 0.66, while the weighted Cohen’s kappa was 0.61 using linear weights and 0.55 using quadratic weights. Given the ordinal nature of the rating scale (low, moderate, high) the weighted kappa provides a more appropriate estimate of agreement and indicates a high level of consistency between the two reviewers, with most disagreements occurring between adjacent categories rather than extreme classifications.

## 11. Limitations

The proposed risk of bias assessment tool for *in vitro* antimicrobial susceptibility testing studies aims to fill the gap in the evaluation of articles included in systematic reviews and meta-analyses. However, our work is not without limitations. This tool was specifically developed for *in vitro* antimicrobial susceptibility testing studies. In these terms, each of the included specific criteria evaluates risk of bias issues specifically concerning the various steps performed in these types of studies.

It is generally accepted that such issues (e.g., methodological issues, measurement performance and accuracy, and reporting issues) may affect various types of *in vitro* studies. However, we think that due to the specificity of the proposed tool, it could not be generally applied in *in vitro* studies other than antimicrobial susceptibility testing ones in the form it is presented in this manuscript. The authors of such studies should adapt the tool as they see fit. In this context, the application of the tool or its adaptations may be assessed for various types of *in vitro* studies in future research.

Furthermore, the lack of performance of replicate experiments and how it affects the scoring of *in vitro* antimicrobial susceptibility testing studies were identified and thoroughly described. According to the proposed tool, all studies in which replicate experiments were not performed were scored as “high risk”. This resulted in the total evaluation of a study as “high risk”. We consider that this is a significant limitation of our tool. However, as it is mentioned above, the presentation of single measurements and not a mean value of multiple measurements, as suggested by CLSI guidelines, poses a high risk of bias for the accuracy of measurements.

Another critical point is the accuracy of the proposed tool. We performed pilot validation of *in vitro* antimicrobial susceptibility testing studies. This included only a small number of studies. In these terms, further studies assessing the performance of the tool in a greater number of articles are needed. Moreover, we estimated and presented the overall inter-reviewer reliability by calculating Cohen’s kappa, unweighted, weighted and quadratic. According to our calculations, the level of agreement is substantial, supporting our belief that the proposed tool presents a relatively high level of accuracy. In this context, we anticipate that future studies will use our tool as a basis and make all necessary improvements in order to further increase its accuracy.

Finally, our validation was only performed regarding the tool proposed for *in vitro* antimicrobial susceptibility testing studies. Thus, apart from the need for adaptation of the proposed tool to other types of *in vitro* studies, further research is necessary in order to validate the application of the tool or its adaptations for the various types of *in vitro* studies.

## 12. Conclusions

We developed a risk of bias assessment tool that may be used in systematic reviews of *in vitro* antimicrobial susceptibility testing studies. The use of this tool will increase the transparency and credibility of such studies.

## Figures and Tables

**Table 1:** Domain-by-domain comparison of risk of bias assessment tools for general *in vitro* studies and their applicability to *in vitro* antimicrobial susceptibility testing studies.

*In Vitro* Antimicrobial Susceptibility Testing-Relevant Domain	Interpretation for *In Vitro* Antimicrobial Susceptibility Testing Studies	INVITES-IN	QUIN	RoBDEMAT
**Methodological standardization**	Adherence to recognized antimicrobial susceptibility testing standards (e.g., CLSI or EUCAST) explicitly described and justified	Not assessed	General methodological rigor only	Not assessed
**Strain selection and representativeness**	Clearly defined inclusion and exclusion criteria Adequately described strain identification Number and diversity of isolates sufficiently reflecting clinical or environmental relevance	Limited (sample description only)	Broad selection criteria	Focused on material testing rather than biological diversity
**Test preparation**	Standardized inoculum preparation/density Validated antimicrobial preparation /dilution Addressed solvent effects	Not assessed	Not assessed	Partially focused on the preparation of materials
**Contamination and cross-contamination control**	Verified isolate purity Performance of aseptic techniques Use of reference strains ans positive/negative controls	Not assessed	Not assessed	Not assessed
**Measurement and observer bias**	Blinded/independent reading of results Calibarated equipment Performance of replicates-repeated testing	Generic observer bias	General outcome assessment	Emphasis on instrument handling
**Reporting and** **publication bias**	All outcomes reported (positive or negative) Available raw data	Assessed	Assessed	Partially assessed
**Interpretive criteria**	Use and reference of standardized interpretive criteria (e.g., CLSI and EUCAST)	Not assessed	Not assessed	Not assessed
**Funding and** **conflicts of interest**	Explicit disclosure of conflicts of interest and funding sources	Assessed	Assessed	Assessed

**Table 2:** Risk of Bias (RoB) Assessment Tool for *In vitro* Antimicrobial Susceptibility Studies.

Domains	Specific Criteria	Score (High, Moderate, Low)
1. Methodological Bias	1.1 Detailed description of the study protocol and adherence to a standardized methodology based on the EUCAST and/or CLSI guidelines for the standard operating procedures for every step 1.2 Use of appropriate statistical models and analyses for the type of data (with justification, if applicable)	
2. Selection Bias	2.1 Explicitly stated strain inclusion and exclusion criteria and selection methods (random or consecutive selection process, or based on availability/convenience/focused with justification) 2.2 Clearly defined and described bacterial strains (e.g., species, source, clinical relevance) 2.3 Number and diversity of isolates (clinical sites, resistance profiles, sample types) adequate and representative of real-world settings–or justification provided (if applicable)	
3. Preparation Bias (including contamination/cross-contamination bias)	3.1 Confirmed purity of isolates and antimicrobials before testing and description of methods used for confirmation 3.2 Detailed description of inoculum preparation, standardization, inoculation, and incubation 3.3 Use of proper solvents and description of dilution methods, detailed preparation, standardization, and storage of stocks 3.4 Report on the implementation of aseptic techniques, use of sterile media and equipment, and contamination control measures-use of positive and negative controls and reference strains for validation	
4. Measurement/Observer Bias	4.1 Independent read of results by two or more observers or blinded reviewers, and results blinded to strain identity and drug tested 4.2 Use of calibrated and validated equipment for the conduction of measurements 4.3 Reported repeat of tests (replicates) with complete documentation of results	
5. Reporting and Publication Bias	5.1 Report on all outcomes and relevant data (including negative results), and deviations–no apparent selective reporting (e.g., omission of failed assays)–justification of missing or excluded data (if applicable) 5.2 Presentation of raw MIC/MBC or report on other relevant data, or availability of Supplementary Data (raw data in Supplementary Files or on open repositories) 5.3 Standard interpretive criteria (e.g., CLSI/EUCAST breakpoints) used and referenced	
6. Bias related to unreported funding and conflicts of interest	6.1 Clearly disclosed funding and conflicts of interest	
**Total**		

## Data Availability

The original contributions presented in this study are included in the article/Supplementary Materials. Further inquiries can be directed to the corresponding author.

## Supplementary Materials

The following supporting information can be downloaded at https://www.mdpi.com/article/10.3390/pathogens15040396/s1.

## Abbreviations

AST	antimicrobial susceptibility testing
ATCC	American Type Culture Collection
CLSI	Clinical and Laboratory Standards Institute
EUCAST	European Committee on Antimicrobial Susceptibility Testing
FDA	Food and Drug Administration
MALDI-TOF	matrix-assisted laser desorption/ionization time-of-flight
MIC	minimum inhibitory concentration
PCR	polymerase chain reaction
PD	pharmacodynamic
PK	pharmacokinetic
RCT	randomized controlled trials
SDD	susceptible dose-dependent
