# Supplementary material for: Proposed Risk of Bias Assessment Tool for In Vitro Antimicrobial Susceptibility Studies
Source: Pathogens. 2026 Apr 7;15(4):396. doi: 10.3390/pathogens15040396 (PMC13119129; doi:10.3390/pathogens15040396)
Supplement: Supplementary file 1 [file pathogens-15-00396-s001.zip › Suppl. file S1. Fully worked example.pdf]

Supplementary File S1: A fully worked example of the domain-level and overall scoring of the proposed risk of bias assessment tool for *in vitro* antimicrobial susceptibility testing studies.

Study assessed: Rollet et al. (2025) Antimicrobial susceptibility of *Clostridioides difficile*. An Argentinian multicenter study of isolates from human patients.

1. Methodological bias

<u>1.1 Detailed description of the study protocol and adherence to a standardized methodology based on the EUCAST and/or CLSI guidelines for the standard operating procedures for every step.</u>

Judgement: Moderate risk of bias

Support for judgment: The antimicrobial susceptibility testing described in the study used and referenced recognized methodological standards (CLSI-M100, 29th edition) for *C. difficile.* However, it remains unclear whether recognized methodological standards regarding the procedures before testing were used.

<u>1.2 Use of appropriate statistical models and analyses for the type of data (with justification, if applicable)</u>

Judgement: Low risk of bias

Support for judgment: Adequately descriptive data analysis (MIC ranges, MIC_{50}/MIC_{90}, resistance proportions). The appropriate statistical models were used and referenced.

Overall scoring for Domain 1: Moderate risk of bias

(≤50% of the criteria in the domain are scored as "moderate" risk of bias, and none as "high" risk of bias)

2. Selection bias

<u>2.1 Explicitly stated strain inclusion and exclusion criteria and selection methods (random or consecutive selection process, or based on availability/convenience/focused with justification)</u>

Judgment: Low risk of bias

Support for judgment: Inclusion criteria were clearly defined. The collection process of isolates involved consecutive patients from multiple centers.

<u>2.2 Clearly defined and described bacterial strains (e.g., species, source, clinical relevance)</u>

Judgment: Low risk of bias

Support for judgment: The *C. difficile* strains, their origin, and their relevance to infection were clearly defined and described.

<u>2.3 Number and diversity of isolates (clinical sites, resistance profiles, sample types) adequate and representative of real-world settings – or justification provided (if applicable)</u>

Judgment: Low risk of bias

Support for judgment: The isolates were collected from consecutive patients from multiple sites and different hospitals.

Overall scoring for Domain 2: Low risk of bias

(all of the criteria in the domain are scored as "low" risk of bias)

3. Preparation Bias (including contamination/cross-contamination bias)

3.1 Confirmed purity of isolates and antimicrobials before testing and description of methods used for confirmation

Judgement: Low risk of bias

Support for judgment: The methods for identification of species, confirmation of isolate purity, and verification of antimicrobial compounds are comprehensively described for suspicious colonies.

3.2 Detailed description of inoculum preparation, standardization, inoculation, and incubation

Judgement: Low risk of bias

Support for judgment: The preparation and standardization of inoculum and the conditions of incubation are described in line with reference standards.

3.3 Use of proper solvents and description of dilution methods, detailed preparation, standardization, and storage of stocks

Judgement: Moderate risk of bias

Support for judgment: Dilution methods are reported. However, the information on solvent preparation and storage conditions of antimicrobial stocks is limited.

3.4 Report on the implementation of aseptic techniques, use of sterile media and equipment, and contamination control measures - use of positive and negative controls and reference strains for validation

Judgement: Moderate risk of bias

Support for judgment: The use of quality control strains is reported. However, contamination control measures and negative controls are not described in detail.

Overall scoring for Domain 3: Moderate risk of bias

(≤50% of the criteria in the domain are scored as "moderate" risk of bias, and none as "high" risk of bias)

4. Measurement / Observer Bias

4.1 Independent read of results by two or more observers or blinded reviewers, and results blinded to strain identity and drug tested

Judgement: High risk of bias

Support for judgment: There is no report of blinded or independent reading of MIC results, which may introduce observer bias.

4.2 Use of calibrated and validated equipment for the conduction of measurements

Judgement: Low risk of bias

Support for judgment: The use of standardized laboratory equipment consistent with EUCAST/CLSI procedures, implying calibration and validation, was not referenced. The reference on the performance of antimicrobial susceptibility testing by the research laboratory at the Facultad de Medicina of the Fundación Barceló could support the use of validated instruments. In this context, the risk of bias is considered low.

4.3 Reported repeat of tests (replicates) with complete documentation of results

Judgement: High risk of bias

Support for judgment: Repeat testing is not clearly documented, and the number of replicates is not explicitly reported.

Overall scoring for Domain 4: High risk of bias

(at least one criterion in the domain is scored as "high" risk of assessment)

5. Reporting and Publication Bias

5.1 Report on all outcomes and relevant data (including negative results), and deviations – no apparent selective reporting (e.g., omission of failed assays) – justification of missing or excluded data (if applicable)

Judgement: Moderate risk of bias

Support for judgment: Susceptibility results for all tested antimicrobials are reported, with no obvious selective omission.

5.2 Presentation of raw MIC/MBC or report on other relevant data, or availability of supple-mentary data (raw data in supplementary files or on open repositories)

Judgement: Moderate risk of bias

Support for judgment: MIC ranges and MIC_{50}/MIC_{90} are reported. However, full isolate-level raw MIC data are not available as supplementary data.

5.3 Standard interpretive criteria (e.g., CLSI/EUCAST breakpoints) used and referenced

Judgement: Low risk of bias

Support for judgment: EUCAST and/or CLSI breakpoints are referenced and used appropriately.

Overall scoring for Domain 5: High risk of bias

(>50% of the criteria in the domain are scored as "moderate" risk of bias)

6. Bias related to unreported funding and conflicts of interest

6.1 Clearly disclosed funding and conflicts of interest

Judgement: Low risk of bias

Support for judgment: The article includes a funding statement and conflicts of interest declaration.

Overall scoring for Domain 6: Low risk of bias

(all of the criteria in the domain are scored as "low" risk of bias)

Overall risk of bias assessment scoring

**Domain**	**Risk**
Domain 1	Moderate
Domain 2	Low
Domain 3	Moderate
Domain 4	High
Domain 5	High
Domain 6	Low
**Overall scoring**	**High** (at least one of the domains is scored as "high" risk of bias)
